# Primary health care challenge for nursing professionals: a narrative review

**DOI:** 10.11606/s1518-8787.2021055002719

**Published:** 2021-12-01

**Authors:** Erika Yurley Duran-Niño, María Stella Campos de Aldana, Ligia Betty Arboleda de Pérez

**Affiliations:** I Universidad de Santander Facultad de Ciencias Médicas y de la Salud Instituto de Investigación Masira Bucaramanga Colombia Universidad de Santander. Facultad de Ciencias Médicas y de la Salud. Instituto de Investigación Masira. Bucaramanga, Colombia

**Keywords:** Atención Primaria de Salud, Educación Basada en Competencias, Estrategias, Enfermería

## Abstract

**OBJECTIVE:**

Carry out a narrative review of scientific literature on nursing professionals training in primary health care (PHC), which allows us to know the challenges facing the renewal of this strategy.

**METHODS:**

Review of the literature found, selection of 55 articles from various scientific sources in the last 10 years in electronic databases (MEDLINE, IME, LILACS, *Centro Cochrane Iberoamericano*, Embase, CUIDEN, CINAHL, BDIE). The selected articles were submitted to an interpretation, synthesis, and critical analysis process for the purpose of selection.

**RESULTS:**

Higher education institutions in Colombia have been working on training of undergraduate students responding to the axes established in the strengthening of human resources in health. This training is used for the application of the integral health care model, focusing on the strengthening of work profiles and the acquisition of competencies that can generate significant contributions to the reality and needs of the individual, family, community, as well as to the different cultures and ethnic groups with humanization, under the general social security health system. An analysis of renewed and comprehensive PHC approaches has been carried out in different countries involving transformations in education with training by competencies, interprofessional work, teamwork, strengthening communication with the community, and health team.

**CONCLUSIONS:**

Based on the narrative review, this research highlights the importance of developing studies on interventions carried out by students with acquired competencies in their training process and management to improve health conditions of the community.

## INTRODUCTION

In Colombia, interest in incorporating primary health care (PHC) into care models has increased after several decades of awareness on the importance of strategies to improve health outcomes of populations. This impulse comes from Law 1438 of 2011 and the Ten-Year Public Health Plan (PDSP - *Plan Decenal de Salud Pública*) of 2012^1,2^to seek strategies that articulate PHC in the system and strengthen human resources in health^3^.

In this context, it is important to know that PHC as part of public health is an approach to health and well-being, strengthening the needs and circumstances of individuals, families and communities, addressing health, physical, mental and social well-being in a comprehensive and interrelated manner^4^. This is a strategy that focuses on improving equity in services so that they are easy to access, and to improve efficiency in the good use of health resources, strengthening the emphasis on prevention in the most vulnerable communities^5^.

The creation of the association for PHC in Colombia^1,2^ was in 2014, for the construction, implementation, monitoring and evaluation of a model of comprehensive care based on PHC^5,6^. Among the priorities of this alliance were the impact on PHC policies and the advancement of capacities and training of human talent for PHC. Subsequently, this partnership for PHC became stronger with the Integrated Health Care Policy (IHCP)^7^.

The creation of the Integrated Health Care Model (IHCM) was in 2016, for the development of the IHCP, to implement services providing health care to the population of a defined territory, in a coordinated manner. The basic units of regulation for the characterization of the population in this model are the integrated health care pathways, which group services and insurers acting according to the health situation analysis (HSA), the Collective Intervention Plan (PIC - *Plan de Intervenciones Colectivas*) and the Benefit Plan^7,8^.

Among the ten components articulating the functions of the IHCM are the importance of strengthening the human resources in health (HRH) in the areas of research, innovation and knowledge appropriation^1^, addressed in the following axes:

Axis 1: HRH Training: The Colombian Ministry of Health and Social Protection^9^(MSPS - *Ministerio de Salud y Protección Social*) will lead the development, updating and strengthening of HRH profiles and competencies, including changes in academic processes that contribute to improving the effectiveness of health care. It will also define the capacities to support the training of Human Talent applied at the undergraduate and postgraduate levels^9^ according to the present needs and in accordance with the IHCP. For this purpose, universities will adjust the curricular plans of the training programs, in order to evaluate the quality and relevance of the processes in the programs, with the support of the Ministry of National Education^9^.

Axis 2: Coordination of human talent in health HRH, in a comprehensive care and service provision scheme: the MSPS will guarantee the implementation of the PHC approach, family and community health, care, comprehensive risk management and the differential approach at the population level, based on the essential functions in public health^9^.

Axis 3: Strengthening of the HRH, responsible for planning and territorial health management: The MSPS oversees the planning and resource management competencies of the model^9^.

Axis 4: The management, planning and progress of HRH working conditions at the national and departmental levels: The MSPS^9^ will define the strategies and starting points for the improvement plans that emerge from the feedback processes.

Thus, the implementation of new policies and their updates have been carried out in the search for the fulfillment of all these objectives, in order for the health systems to adopt them and comply with the regulations.

Based on the above, the importance of the competencies that a health professional should have is clear, since they allow the progressive development of the necessary skills for the implementation of IHCM. Thus, public health competencies are essential, not related to the skills that a given profession must have or perform, but to the skills necessary for the optimal functioning of the different levels of health care^3,9^.

Another important aspect to consider is the Integrated Territorial Action Model (ITAM), whose purpose is to achieve the best health results, seeking to respond to the prioritization of identified health needs by providing quality care.

ITAM is based on PHC, with emphasis in familiar^10^ and communitarian aspects of the regions and populations, based on the different routes of lines of action and coordinating actions with the agents of the health system, in communities and other systems, operating interinstitutionally and with the communities by agreements with the support of the Ministry of Health^10^.

The World Health Organization (WHO) currently defines PHC as follows:

Primary health care is the essential health care accessible to all individuals and families in the community using means acceptable to them, with their full participation and at a cost affordable to the community and the country. It is the core of the country’s health system and is an integral part of the overall socio-economic development of the community.

In the Alma Ata declaration, PHC was defined as: essential health care focused on methods and technologies of the practices, scientifically based and socially accepted, being within the reach of the families of the community, by participation, having a cost that the community and the country are able to manage at each and every stage, having a spirit of self-determination and self-responsibility^11,5^.

At the international level, it is evident that the curriculum for nursing education in Latin America and the Caribbean has included the principles and values of universal health and PHC. Likewise, it has also included the principles that maintain the modalities of education, transforming critical thinking, problem solving, and thus the need to promote a change in the paradigm of nursing education^1,12^.

The different studies developed in relation to these competencies identified the need for training human talent in health, in public health, and PHC competencies in the academic programs of health programs and institutional strengthening for the implementation of the IHCM^1^.

In addition, the survey results highlight that although the programs are implemented in the curricula, the disciplines and specific competencies of the basic and clinical training area do not specify or prioritize essential aspects of public health and PHC^1^. This shows the importance of strengthening the competencies of knowing how to know, knowing how to do and knowing how to be, as well as in the implementation of intersectoral programs, in epidemiological surveillance activities and in public health research, so that they can be put into practice in the professional role^16^.

To achieve alignment with the new comprehensive care model, it is necessary to make a curricular adjustment so that students in the health area acquire the necessary competencies for the successful implementation and articulation of the IHCM; as referred to in two of the competencies of the nursing professional, in order to influence the health of the community and its environment, by projecting care aimed at meeting the needs of the individual, contributing to the construction of a dignified life and general wellbeing^17^. The objective is to guarantee participation in the formulation, design, implementation and control of health and nursing policies, programs, plans and projects, as well as to establish and develop policies and models of nursing care in accordance with national health policies^18^.

The professional competencies required for effective and quality development in health involve adjustments in the training processes in the education offered by universities. These changes must be a commitment to joint, integrated and symmetrical action by the institutions of the education and health sectors^7,19^.

In support of the above, a study conducted on PHC among nursing professionals showed that 64.6% presented unacceptable knowledge in relation to PHC and only 2.6% achieved a better score. There were weaknesses in promotion, prevention, epidemiological surveillance and environmental control activities. Academies should reconsider the nursing curriculum, so that its contents and activities in PHC promote skills and tools related to community participation^20^.

The department of Santander leads the execution of primary care and the implementation of the new IHCM in Colombia^5^. A study whose main objective was to identify the perception of health workers and their supervisors in first level institutions regarding their competencies to perform within IHCM found that several competencies must be strengthened in order to implement IHCM^21^.

The objective of the research is to carry out a narrative review of the scientific literature that will provide us with information on PHC in nursing professionals, allowing us to know the challenges before the renewal of this strategy.

## METHODS

In this narrative review, we collected a series of articles related to PHC from several bibliographic literatures aiming to learn about PHC in nursing professionals and the challenges before the renewal of strategies for PHC. This will allow us to achieve health promotion, ensuring equity in care, interaction with the interprofessional and intersectoral team and the diversity of services in accordance with Law 3280, complementing the different levels of care and services in a coordinated manner and with competencies to strengthen and develop health systems based on PHC^22^.

### Sample

Search for articles in databases: *Literatura Latinoamericana y del Caribe en Ciencias de la Salud* (LILACS), IME, MEDLINE, *Centro Cochrane Iberoamericano*, BDIE, Cumulative Index to Nursing and Allied Health Literature (CINAHL), *Base de Datos Bibliográfica sobre Cuidados de Salud en Iberoamérica* (CUIDEN), Embase, during the first half of 2019, published between 2009 and 2019, written in Spanish. Fifty-five potential studies^23^ “*Atención primaria en salud en los profesionales de enfermería*” (Primary health care in nursing professionals) were found, of which 40 were included in the review. The Figure shows the most relevant articles selected in the narrative review of the literature. The choice was by convenience, considering the different inclusion criteria. These included the following: scientific research articles and research should be related to the topic “*Atención primaria en salud en los profesionales de enfermería*” (Primary health care in nursing professionals). The exclusion criteria were: articles not found in the selected databases and bibliography that lacks the review topic. This article is in the framework of the research project “*Ruta de formación de competencias en salud pública y atención primaria en salud, en el Programa de Enfermería de la Universidad de Santander – Fase I*”. In the following are the definitions of the descriptors used for this review.

**Figure:**
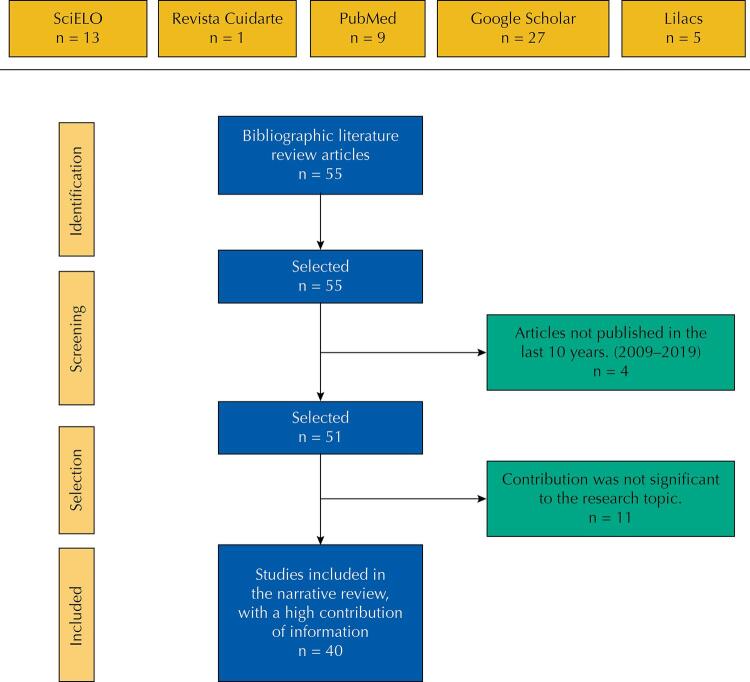
Selection process of Cochrane systematic review articles.

### Analysis Plan

Based on the principles established by the PRISMA declaration, we proceeded to review and select articles from all the databases that met the necessary requirements for our narrative review, resulting in 55 articles: Scientific Electronic Library Online – SciELO (13), *Revista Cuidarte* (1), PubMed (9), Google Scholar (27), Lilacs (5), reaffirming the inclusion criteria and the relevance of the articles for the proposed objectives.

The first step consisted of eliminating the articles not corresponding to the years 2009–2019: 4 articles. After reading the title and abstract, we excluded 11 articles. Of the resulting articles, 51 were read in full text, selecting the articles that formed this work according to the established inclusion criteria^19^. At the end of the process, we included 40 articles in the narrative review. The Figure shows a PRISMA^24^ diagram, according to the selection process of the Cochrane Articles systematic reviews in order to discard the articles that were useless to us.

A data base with 55 articles were collected was constructed; 10 were reviewed per week in order to obtain more information and thus give precision to the proposal. We then made a selection of articles that met the necessary requirements for our narrative review, obtaining 40 articles, reaffirming the inclusion criteria and the relevance of the articles and documents for the proposed objectives.

### Ethical Aspects

The project is a narrative review, with no risk to humans as it uses only published articles. In this respect, it is in accordance with Resolution 8430 of 1993 of the MSPS and with the Declaration of Helsinki. In addition, we respected everything concerning copyright established in Law 23 of 1982.

## RESULTS

Previous studies showed the importance of including in the curricula of health programs some fundamental aspects for the adequate implementation of IHCM and now ITAM. These aspects include leadership development, autonomy of health workers, intersectoral coordination skills and interdisciplinary work^5^, with a contextualization of the population and its territory.

Currently, higher education institutions in Colombia are working on the training of undergraduate students, responding to the axes established in the strengthening of human resources in health for the implementation of ITAM. It focuses on strengthening work profiles and acquiring competencies to generate significant contributions to the reality and needs of the individual, family, and community. As well as the consolidation of different cultures and ethnicities by humanization, benefiting from the general system of social security in health, which allows prioritizing the care provided integrally, with quality and excellence, by the nursing professional in collective health area, complemented by interprofessional work.

The gaps between the results with the characteristics of the training and the performance needs have been strengthening with the new educational approach by competencies for health promotion, disease prevention and palliative care, redirecting the profiles of equitable training at hospital and community level.

## DISCUSSION

WHO reaffirms the importance of PHC principles and values, selecting solidarity, social justice, equity and universal access, interprofessional and community participation. The elements, policies, primary models employed as a health system strategy for health care are ITAM, IHCM, IHCP, accountability and sustainability, health equit^25^.

Thus, PHC requires a competency-based interprofessional team with cognitive and operative training to work as a team. It is also necessary to have leadership and training to make health decisions. Strengthening communication is also required, responding to the health needs of the population in terms of appropriate and equitable health^28^.

## CONCLUSIONS

The general social security health system considers PHC as the priority strategy, emphasizing health promotion and disease prevention. It includes essential elements in the orientation towards quality with timely response to the health needs of the population, with comprehensive and continuous care offered by the nursing professional trained with competencies and based on the existing regulations, improving the quality of life and equity, reviving and renewing PHC as the central axis in the health system.

The studies reviewed and analyzed consider the acquisition of competencies in the role of nurses in health care as an important issue. It is very important to adhere to the policies (IHCP), health care models (IHCM) and the primary objective: the approach to families and communities (ITAM) accessible to the entire population.

Nursing education worldwide and in Latin America and the Caribbean requires within its formation, in addition to the principles of transformative education, the development of critical and complex thinking as modalities of education. A kind of education capable to face and provide normative and assistance solutions to health development, particularly in different sectors, being the health sector a critical point since it is in charge of maintaining and improving the health of the population^31,32^.

The narrative review highlights the importance of developing research studies on the interventions carried out by students with the competencies acquired in their training process. The review also shows how has been the management to improve health conditions of the community and to determine the needs faced by families and communities at international, national, departmental and regional levels, favoring timely health care.

In view of the recent changes in the IHCP, the IHCM and the ITAM, there is little bibliographic documentation related to the perception of health students on these topics. Thus, it becomes a challenge for educational institutions to know and apply them in their training practices.

Finally, it is possible to state that nursing science has currently been concerned with graduate training, emphasizing family medicine. However, so far, these opportunities are concentrated in the area of education.
