# Supplementary material for: Primary health care challenge for nursing professionals: a narrative review
Source: Rev Saude Publica. 2021 Dec 1;55:100. doi: 10.11606/s1518-8787.2021055002719 (PMC8664055; doi:10.11606/s1518-8787.2021055002719)
Supplement: Translation

# Translation: Atención primaria en salud reto de los profesionales de enfermería: una revisión narrativa

**Authors:** Erika Yurley Duran-Niño, María Stella Campos de Aldana, Ligia Betty Arboleda de Pérez

**Affiliations:** I Colombia Universidad de Santander. Facultad de Ciencias Médicas y de la Salud. Instituto de Investigación Masira. Bucaramanga, Colombia

**Keywords:** Atención Primaria de Salud, Educación Basada en Competencias, Estrategias, Enfermería

## Abstract

**OBJETIVO:**

Realizar una revisión narrativa de la literatura científica sobre la formación en la atención primaria en salud (APS) en los profesionales de enfermería permitiéndonos conocer los retos ante la renovación de esta estrategia.

**MÉTODOS:**

Se realizó revisión de la literatura encontrada seleccionando 55 artículos de varias fuentes científicas, entre los últimos 10 años, en bases de datos electrónicas (MEDLINE, IME, LILACS, Centro Cochrane Iberoamericano, Embase, CUIDEN, CINAHL, BDIE); la selección se realizó mediante la interpretación, síntesis y análisis crítico de los artículos seleccionados.

**RESULTADOS:**

Las instituciones de educación superior en Colombia vienen trabajando en la formación de los estudiantes de pregrado, respondiendo a los ejes establecidos en el fortalecimiento del recurso humano en salud. Esto, para la aplicación del modelo integral de atención en salud, enfocándolo al fortalecimiento de los perfiles laborales y adquisición de competencias que puedan generar aportes significativos a la realidad y necesidades de individuo, familia, comunidad, así como las diferentes culturas y grupos étnicos con humanización, acogiéndose al sistema general de seguridad social en salud. En diferentes países se ha realizado análisis de los enfoques de APS renovada e integral, implicando transformaciones en la educación con la formación por competencias, trabajo interprofesional, trabajo en equipo, fortaleciendo la comunicación con la comunidad y equipo de salud.

**CONCLUSIONES:**

Basado en la revisión narrativa, se resalta la importancia de desarrollar estudios investigativos de cómo han sido las intervenciones realizadas por los estudiantes con las competencias adquiridas en su proceso formativo y la gestión para mejorar las condiciones de salud de la comunidad.

## INTRODUCCIÓN

En Colombia se ha aumentado el interés al incorporar la atención primaria en salud (APS) dentro de los modelos de atención después de conocerse por varias décadas de la importancia de las estrategias para mejorar los resultados en salud de las poblaciones. Este impulso viene dado desde la Ley 1438 de 2011 y el Plan Decenal de Salud Pública (PDSP) de 2012^1,2^ para buscar estrategias que articulen la APS en el sistema y fortalezcan el recurso humano en salud^3^.

Dentro de este contexto es importante saber que la APS como parte de la salud pública se entiende como enfoque para la salud y el bienestar, fortaleciendo las necesidades y circunstancias de las personas, las familias y comunidades, abordando la salud, el bienestar físico, mental y social de forma global e interrelacionada^4^. Esta es una estrategia que va enfocada y gira en torno a mejorar la igualdad en los servicios para que sean de fácil acceso, y para mejorar la eficiencia en el buen uso de los recursos de salud, logrando fortalecer el énfasis en la prevención en las comunidades más vulnerables^5^.

En el año 2014 se conformó la asociación por la APS en Colombia^1,2^, para la construcción, ejecución, seguimiento y evaluación de un modelo de atención integral basada en APS^5,6^. Dentro de las prioridades de esta alianza se planteó la incidencia en las políticas sobre la APS y el avance de las capacidades y el entrenamiento del talento humano para la APS. Posteriormente, esta alianza por la APS se fortaleció con la Política de Atención Integral en Salud (PAIS)^7^.

En el año 2016, para el desarrollo de la PAIS se conformó el Modelo Integral de Atención en Salud (MIAS) con el objetivo de implementar los servicios brindando cuidado en la salud de la población de un territorio definido, de manera coordinada. Las unidades básicas de regulación en donde se debe caracterizar a la población en este modelo se llaman rutas integrales de atención en salud y agrupan servicios y aseguradores que actúan Según el análisis de la situación de salud (ASIS), el Plan de Intervenciones Colectivas (PIC) y el Plan de Beneficios^7,8^.

Dentro de los diez componentes que articulan las funciones del MIAS se encuentra: la importancia de fortalecer el recurso humano en salud (RHS) en áreas de la investigación, innovación y apropiación del conocimiento^1^; abordado en los siguientes ejes:

Eje 1: La Formación del RHS: El Ministerio de Salud y Protección Social (MSPS)^9^ liderará el desarrollo, actualización y fortalecimiento de los perfiles y competencias del RHS, comprendiendo cambios en los procesos académicos que contribuyan a mejorar la resolutividad en la atención en salud. También definirá las capacidades para apoyar la formación del Talento Humano aplicado en pregrado y posgrado^9^ de acuerdo a las necesidades presentes y acorde a la PAIS; para ello, se ajustarán los planes curriculares de los programas de formación por parte de las universidades, donde la calidad y pertinencia de los procesos se evaluaran en los programas, con el acompañamiento del Ministerio de Educación Nacional^9^.

Eje 2: La coordinación del talento humano en salud RHS, en esquema de cuidado integral y provisión de servicios: el MSPS garantizará la implementación en el enfoque de APS, salud familiar y comunitaria, el cuidado, la gestión integral del riesgo y el enfoque diferencial a nivel poblacional, teniendo como base las funciones esenciales en salud pública^9^.

Eje 3: El fortalecimiento del RHS, responsable de la planeación y gestión territorial en salud: El MSPS supervisa las competencias de planeación y gestión de los recursos del modelo^9^.

Eje 4: La gestión, planificación y progreso de condiciones laborales del RHS a nivel nacional y departamental: El MSPS^9^ definirá las estrategias y los momentos de inicio de los planes de mejora que surjan de los procesos de retroalimentación.

Así, en la búsqueda del cumplimiento de todos estos objetivos se han venido implementando nuevas políticas y actualizaciones de las mismas con el fin de que los sistemas de salud las adopten y den cumplimiento a la normatividad.

Con base en lo anterior, queda clara la importancia de las competencias que debe tener un profesional de la salud, una vez que permiten el desarrollo progresivo de las habilidades necesarias para la implementación del MIAS. De esta manera, son esenciales las competencias en salud pública, no relacionadas a las capacidades que debe tener o desempeña determinada profesión, sino relativas a las capacidades necesarias para el óptimo funcionamiento de los diferentes niveles de atención en salud^3,9^.

Otro aspecto importante a tener en cuenta es el Modelo Integral Territorial (MAITE), cuyo propósito es alcanzar los mejores resultados en salud, buscando responder a la priorización de necesidades en salud identificadas brindando atención de calidad.

El MAITE se fundamenta en la APS, con énfasis familiar^10^ y comunitario en las regiones y poblaciones, basado en las rutas diferentes de líneas de acción y coordinando acciones con los agentes del sistema de salud, en comunidades y otros sistemas, operando interinstitucionalmente y con las comunidades mediante acuerdos con apoyo del Ministerio de Salud^10^.

Actualmente la Organización Mundial de la Salud (OMS) define la APS del siguiente modo:

La atención primaria de salud es la asistencia sanitaria esencial accesible a todos los individuos y familias de la comunidad a través de medios aceptables para ellos, con su plena participación y a un costo asequible para la comunidad y el país; es el núcleo del sistema de salud del país y forma parte integral del desarrollo socio-económico general de la comunidad.

En la declaración de Alma Ata, se definió la APS como: asistencia sanitaria esencial enfocada en métodos y tecnologías en las prácticas, fundamentada científicamente y aceptada socialmente, siendo del alcance de las familias de la comunidad, por medio de la participación, teniendo un costo que la comunidad y el país son capaces de sobrellevar en todas y cada una de las etapas, teniendo espíritu de auto determinación y de auto responsabilidad^11,5^.

A nivel internacional se puede evidenciar que el plan de estudio para la formación educativa en enfermería en América Latina y el Caribe ha acogido los principios y valores de la salud universal y la APS; así mismo, ha acogido también los principios que mantienen las modalidades de educación, transformando el pensamiento crítico, la solución de problemas, y con ello la necesidad de promover un cambio en el paradigma de la educación en enfermería^1,12^.

Los diferentes estudios que se han desarrollado frente a estas competencias han identificado la necesidad de formación del talento humano en salud en competencias de salud pública y de APS en los programas académicos de los programas de salud y el fortalecimiento institucional para la implementación del MIAS^1^.

Sumado a lo anterior, los resultados de la investigación recalcan que si bien los programas que implementan en los currículos, las asignaturas y competencias específicas al área de formación básica y clínica, no especifican o no priorizan aspectos esenciales de la salud pública y de la APS^1^. Eso evidencia la importancia del fortalecimiento de las competencias del saber conocer, saber hacer y el saber ser, así como en la ejecución de programas intersectoriales, en las actividades de vigilancia epidemiológica y en la investigación en salud pública, con el fin de se puedan poner en práctica en su rol de profesional^16^.

Para lograr una consonancia con el nuevo modelo de atención integral, se encuentra necesario realizar un ajuste curricular para que los estudiantes del área de la salud adquieran las competencias necesarias para la implementación y articulación exitosa del MIAS¸ como se hace referencia en dos de las competencias del profesional de enfermería, con el fin de incidir en la salud de la comunidad y su entorno, por medio de la proyección del cuidado direccionado a la satisfacción de las necesidades del individuo, contribuyendo a la construcción de una vida digna y bienestar general^17^. Eso tiene el objetivo de garantizar la participación en la formulación, diseño, implementación y control de las políticas, programas, planes, y proyectos de salud y enfermería, así como de establecer y desarrollar políticas y modelos de cuidado de enfermería en concordancia con las políticas nacionales de salud^18^.

Las competencias profesionales solicitadas para el desarrollo efectivo y con calidad en salud exigen ajustes en los procesos formativos en la educación ofrecida por las universidades; esos cambios deben ser un compromiso de acción conjunta, integrada y simétrica de las instituciones de los sectores de educación y de salud^7,19^.

Apoyando lo anterior, un estudio realizado desde la APS en los profesionales de enfermería arrojó que el 64,6% presentó un conocimiento no aceptable con relación a APS y observó que solo el 2,6% logró una mejor puntuación; se evidenciaron debilidades en actividades de promoción, prevención, vigilancia epidemiológica y control ambiental. Desde las academias se debe replantear el currículo de enfermería, para que sus contenidos y actividades en APS fomenten las capacidades y herramientas relacionadas a la participación comunitaria^20^.

El departamento de Santander encabeza en la ejecución en la atención primaria y lidera la implementación del nuevo MIAS en Colombia^5^; un estudio realizado cuyo objetivo principal era identificar la percepción de los trabajadores de salud y sus supervisores en instituciones de primer nivel respecto a sus competencias para desempeñarse dentro del MIAS encontró que varias competencias se deben fortificar para poder implementar el MIAS^21^.

El objetivo de la investigación es realizar una revisión narrativa de la literatura científica que nos dé información sobre APS en los profesionales de enfermería, permitiéndonos conocer los retos ante la renovación de esta estrategia.

## MÉTODOS

En la presente revisión narrativa se recolectaron una serie de artículos relacionados con APS de varias literaturas bibliográficas teniendo como objetivo conocer la APS en los profesionales de enfermería y los retos ante la renovación de estrategias para la APS. Esto nos permitirá logros con la promoción de la salud, garantizando la equidad en la atención y la interacción con el equipo interprofesional e intersectorial y la diversidad de servicios acorde a la Ley 3280, complementándose en los diferentes niveles de atención y servicios de manera coordinada y con competencias para fortalecer y desarrollar sistemas de salud basados en la APS^22^.

### Muestra

Búsqueda de artículos en las bases de datos: Literatura Latinoamericana y del Caribe en Ciencias de la Salud (LILACS), IME, MEDLINE, Centro Cochrane Iberoamericano, BDIE, *Cumulative Index to Nursing and Allied Health Literature* (CINAHL), Base de Datos Bibliográfica sobre Cuidados de Salud en Iberoamérica (CUIDEN), Embase, durante el primer semestre de 2019, publicados entre los años 2009 y 2019, escritos en español. Se encontraron 55 estudios potenciales^23^ “Atención primaria en salud en los profesionales de enfermería”, de los cuales se incluyeron 40 estudios con los que se adelantó la revisión. En la Figura se muestran los artículos más relevantes seleccionados en la revisión narrativa de la literatura; la elección se realizó por conveniencia, teniendo en cuenta los diferentes criterios de inclusión. Dentro de estos se plantearon los siguientes: artículos de investigación científica e investigación debe ser referente al tema “Atención primaria en salud en los profesionales de enfermería”; en cuanto a los criterios de exclusión, se determinaron: artículos que no se encuentren en las bases de datos seleccionadas y bibliografía que no aborde el tema de revisión. Este artículo se encuentra en el marco del proyecto de investigación “Ruta de formación de competencias en salud pública y atención primaria en salud, en el Programa de Enfermería de la Universidad de Santander – Fase I”; a continuación, se muestra la definición de los descriptores utilizados para esta revisión.

**Figura:** Proceso de selección de los artículos Cochrane revisiones sistemáticas.

### Plan de Análisis

A partir de los principios establecidos por la declaración PRISMA, se procedió a hacer una revisión y selección de artículos de entre todas las bases de datos que cumplieron con los requisitos necesarios para nuestra revisión narrativa, que obtuvo un total de 55 artículos: *Scientific Electronic Library Online* – SciELO (13), Revista Cuidarte (1), PubMed (9), Google Académico (27), Lilacs (5), reafirmando los criterios de inclusión y la relevancia de los artículos para los objetivos propuestos.

El primer paso consistió en la eliminación de los artículos que no correspondían a los años entre 2009–2019: un total de 4. Después de la lectura del título y el resumen, se excluyeron 11 artículos; de los artículos resultantes, en 51 se realizó la lectura del texto completo, filtrando los artículos que dieron forma a este trabajo a raíz de los criterios de inclusión establecidos^19^. Al final del proceso, 40 artículos se incluyeron en la revisión narrativa. En la Figura se puede observar un diagrama PRISMA^24^, según proceso de selección de los Artículos Cochrane revisiones sistemáticas para poder descartar los artículos que no nos servían.

Se construyó una base de datos donde se recolectaron 55 artículos; se revisaron 10 por semana con el fin de obtener más información y así dar claridad a lo propuesto. Luego se procedió a hacer una selección de artículos que cumplieron con los requisitos necesarios para nuestra revisión narrativa, de los que se obtuvo un total de 40 artículos, reafirmando los criterios de inclusión y la relevancia de los artículos y documentos para los objetivos propuestos.

### Aspectos Éticos

El proyecto es una Revisión Narrativa; por tanto, no representa un riesgo para los humanos porque no se realiza sobre individuos sino sobre artículos publicados. En ese sentido, está de acuerdo con la Resolución 8430 de 1993 del MSPS y con la Declaración de Helsinki. Adicionalmente, se respeta todo lo concerniente a los derechos de autor establecido en la Ley 23 de 1982.

## RESULTADOS

Estudios previos han demostrado la importancia de incluir en los currículos de los programas de salud algunos aspectos fundamentales para la adecuada implementación del MIAS y ahora el MAITE. Estos aspectos incluyen el desarrollo del liderazgo, la autonomía de los trabajadores de la salud, las habilidades de coordinación intersectorial y trabajo interdisciplinar^5^, con una contextualización de la población y su territorio.

Actualmente las instituciones de educación superior en Colombia vienen trabajando en la formación de los estudiantes de pregrado, respondiendo a los ejes establecidos en el fortalecimiento del recurso humano en salud para la aplicación del MIAS. Se enfoca el fortalecimiento de los perfiles laborales y adquisición de competencias que puedan generar aportes significativos a la realidad y necesidades de individuo, familia, comunidad, así como las diferentes culturas y grupos étnicos con humanización, acogiéndose al sistema general de seguridad social en salud, lo cual permite priorizar el cuidado, brindado integral con calidad y excelencia en la atención por el profesional de enfermería en el área de la salud pública complementado con el trabajo interprofesional.

Las brechas entre los resultados con las características de la formación y las necesidades del desempeño se han venido fortaleciendo con el nuevo enfoque educativo por competencias para la promoción de la salud, la prevención de la enfermedad y el cuidado paliativo, re direccionando los perfiles de formación equitativa a nivel hospitalario y comunitario.

## DISCUSIÓN

La OMS reafirma la importancia de los principios y valores de la APS, seleccionando la solidaridad, justicia social, equidad y acceso universal, interprofesional y participación comunitaria. Los elementos, políticas, modelos primordiales empleados como estrategia del sistema de salud para la atención en salud son MAITE, MIAS, PAIS, responsabilidad y sostenibilidad, equidad en salud^25^.

Es así como en la APS se requiere un equipo interprofesional formado basado en competencias con formación cognitiva y operativa para trabajar en equipo; se requiere también tener liderazgo y formación para tomar decisiones en salud; también es necesario el fortalecimiento de la comunicación, respondiendo a las necesidades de salud y de la población en tema de salud oportuna y equitativa^28^.

## CONCLUSIONES

El sistema general de seguridad social en salud considera la APS como la estrategia prioritaria, haciendo énfasis en promoción de la salud y prevención de la enfermedad. Incluye elementos esenciales en la orientación hacia la calidad con respuesta oportuna a las necesidades de salud de la población, mediante una atención integral y continua ofrecida por el profesional de Enfermería formado con competencias y basado en la normatividad existente, mejorando la calidad de vida y la equidad, resurgiendo y renovando la APS como eje central en el sistema sanitario.

Los estudios revisados y analizados consideran de interés la adquisición de competencias en el rol que desempeñan las enfermeras para la atención en salud siendo de gran importancia acogerse a las políticas (PAIS), modelos de atención en salud (MIAS) y al objetivo primordial: el abordaje a familias y comunidades (MAITE) que sea asequible a toda la población.

La educación a nivel mundial y de América Latina y el Caribe en enfermería requiere dentro de su formación, además de los principios de la educación transformadora, el desarrollo del pensamiento crítico y complejo como modalidades de educación, con capacidad de afrontar y dar soluciones normativas y asistenciales al desarrollo en salud particularmente y en diferentes sectores, siendo el de salud un punto crítico pues es el encargado del mantenimiento y mejoramiento de la salud de la población^31,32^.

Basado en la revisión narrativa, se resalta la importancia de desarrollar estudios investigativos de cómo han sido las intervenciones realizadas por los estudiantes con las competencias adquiridas en su proceso formativo y cómo ha sido la gestión para mejorar las condiciones de salud de la comunidad y para determinar las necesidades que afrontan las familias y comunidades a nivel internacional, nacional, departamental y regional, favoreciendo la oportuna atención en salud. Frente a los recientes cambios en la PAIS, el MIAS y el MAITE, se evidencia poca documentación bibliografía relacionada a la percepción de los estudiantes del área de la salud sobre estas temáticas; así, se convierte en un reto para las instituciones educativas el conocimiento y aplicación en sus prácticas formativas.

Finalmente, puede decirse que hoy en día la ciencia de enfermería se ha preocupado de la formación de postgrados con énfasis en medicina familiar pero que hasta ahora se están dando estas oportunidades en educación.
